# Clinical decision support improves physician guideline adherence for laboratory monitoring of chronic kidney disease: a matched cohort study

**DOI:** 10.1186/s12882-015-0159-5

**Published:** 2015-10-15

**Authors:** Jennifer Ennis, Daniel Gillen, Arthur Rubenstein, Elaine Worcester, Mark E. Brecher, John Asplin, Fredric Coe

**Affiliations:** Litholink Corporation®, A LabCorp Company, Chicago, IL USA; University of California at Irvine, Irvine, CA USA; University of Pennsylvania, Philadelphia, PA USA; University of Chicago, Chicago, IL USA; Laboratory Corporation of America® Holdings, Burlington, NC USA

## Abstract

**Background:**

Guidelines exist for chronic kidney disease (CKD) but are not well implemented in clinical practice. We evaluated the impact of a guideline-based clinical decision support system (CDSS) on laboratory monitoring and achievement of laboratory targets in stage 3–4 CKD patients.

**Methods:**

We performed a matched cohort study of 12,353 stage 3–4 CKD patients whose physicians opted to receive an automated guideline-based CDSS with CKD-related lab results, and 42,996 matched controls whose physicians did not receive the CDSS. Physicians were from US community-based physician practices utilizing a large, commercial laboratory (LabCorp®).

We compared the percentage of laboratory tests obtained within guideline-recommended intervals and the percentage of results within guideline target ranges between CDSS and non-CDSS patients. Laboratory tests analyzed included estimated glomerular filtration rate, plasma parathyroid hormone, serum calcium, phosphorus, 25-hydroxy vitamin D (25-D), total carbon dioxide, transferrin saturation (TSAT), LDL cholesterol (LDL-C), blood hemoglobin, and urine protein measurements.

**Results:**

Physicians who used the CDSS ordered all CKD-relevant testing more in accord with guidelines than those who did not use the system. Odds ratios favoring CDSS ranged from 1.29 (TSAT) to 1.88 (serum phosphorus) [CI, 1.20 to 2.01], *p* < 0.001 for all tests. The CDSS impact was greater for primary care physicians versus nephrologists. CDSS physicians met guideline targets for LDL-C and 25-D more often, but hemoglobin targets less often, than non-CDSS physicians. Use of CDSS did not impact guideline target achievement for the remaining tests.

**Conclusions:**

Use of an automated laboratory-based CDSS may improve physician adherence to guidelines with respect to timely monitoring of CKD.

**Electronic supplementary material:**

The online version of this article (doi:10.1186/s12882-015-0159-5) contains supplementary material, which is available to authorized users.

## Background

Many studies have shown that, as a rule, physicians do not follow clinical guidelines very well [1–3]. Common reasons include lack of awareness, familiarity, and agreement, inertia of previous practice, and external barriers such as the guidelines being inconvenient, confusing, and cumbersome [1]. These facts have been important in driving the development of clinical decision support systems (CDSS) that convey guideline materials to physicians in ways that are convenient and therefore potentially effective in altering behavior [4].

However, CDSS have not consistently improved physician adherence to guidelines concerning proper and timely test ordering [5–9]. Although some trials have shown positive results, many of them were conducted within healthcare systems whose structures helped implement the recommendations [9]. Thus far, no trials or studies have documented the effects of a scalable CDSS like the one presented here which depends upon nothing but algorithm-driven advice embedded in aboratory reports and sent to physicians as part of their routine work flow.

Because of manifest necessity for achieving proper care of patients with chronic kidney disease (CKD), extensive guidelines have been created by the National Kidney Foundation Kidney Disease Outcomes Quality Initiative (KDOQI™) [10–14] and the Kidney Disease: Improving Global Outcomes (KDIGO®) Work Groups [15–19]. To-date, few CDSS have attempted to implement them [20–23]. We present here the results of a matched cohort study of a CDSS designed to implement the KDOQI™/KDIGO® guidelines via laboratory-based interpretive reports delivered to community-based physicians as part of their routine laboratory reporting from a single national testing vendor. Despite the absence of any organized research or practice structures to facilitate adherence to guidelines, we were able to show an improved alignment between guideline recommendations and both test ordering and, in two instances, test results.

## Methods

### The CDSS program

The CDSS program is an enhanced laboratory reporting system serving physicians who use a large commercial laboratory (Laboratory Corporation of America® Holdings (LabCorp)). KDOQI™ and KDIGO® guidelines concerning kidney function, CKD mineral bone disorder, anemia, and lipids were translated into a comprehensive reporting program by some of the authors (FC, JA, EW, and JE). Guideline translation and adjudication of differences between guidelines were supervised by a panel of authorities, many of whom had participated in their development (Additional file 1). For each of these four areas, the reporting program interprets an individual patient’s current laboratory results and changes over time to produce suggestions for treatment and follow up testing (Additional file 2). The interpretive text is part of each laboratory report.

The CDSS program also includes optional flow sheets of all relevant laboratory results, selected graphs with trend lines (Additional files 3 and 4), ‘patient friendly’ explanations of laboratory test results, and dietary guides for sodium, phosphorus, potassium, and protein restriction (Additional files 5 and 6).

The LabCorp sales force presented the CDSS program to physicians as an optional service at no additional cost to the patient or physician. “CDSS physicians” were those who chose to receive any component of the program offering. “Control physicians” either were not offered CDSS or were offered it but chose not to receive it. The physicians who chose to use our CDSS practice practice in 32 US states. The practices range from solo physicians to very large multi-specialty groups that use LabCorp as a laboratory provider.

### Study design

We chose a matched cohort study design to control for potential confounding and selection bias. Western Institutional Review Board® (Olympia, WA) approved the study protocol and determined that the study met requirements for waiver of consent and waiver of authorization for use and disclosure of protected health information on August 24, 2012.

We identified 14,524 ‘CDSS patients’ 18 years or older whose physicians were CDSS physicians and who had at least two estimated glomerular filtration rate (eGFR) results between 15 and 59 ml/min/1.73 m^2^ at least three months apart (CKD stage 3 or 4). eGFR is calculated using the CKD-EPI equation and reported with all serum creatinine measurements at LabCorp. Serum creatinine is traceable to the isotope dilution mass spectroscopy (IDMS) standard. Race, used in computing eGFR, was not available for the overwhelming majority (approximately 96 %) of patients. When race was unknown, the eGFR calculation for a non-African American patient was used by default because the majority of the US population is non-African American. Physicians received both eGFR calculations as part of their standard laboratory results report. All patients had a first laboratory order (accession) between April 2009 and June 2012. The date of the last accession was June 2013. Median follow-up time was 0.73 years. The short median follow up interval reflects that the majority of providers were enrolled during 2011–2012.

Non-CDSS patients (‘controls’) were matched in a four to one ratio when possible on age (± five years), gender, eGFR (±5 ml/min/1.73 m^2^), zip code (or state if zip code was not available), time of the initial eGFR result of the CDSS patient, and the type of ordering physician (primary care, nephrology, or other specialty). The final analysis dataset consisted of 12,353 CDSS patients and 42,996 matched controls: 9469 CDSS patients with four controls, 678 CDSS patients with three controls, 880 CDSS patients with two controls, and 1326 CDSS patients with a single control. There were 1740 unique CDSS physicians and 14,402 unique control physicians in the analysis. The majority of physicians (60 and 81 % of CDSS and control physicians, respectively) contributed one to three patients to the study. We could not match on providers as well as patients because of limited information concerning provider details.

### Definition of study endpoints

We considered 11 analytes: eGFR, plasma parathyroid hormone (PTH), serum calcium, phosphorus, 25-hydroxy vitamin D (25-D), total carbon dioxide (CO_2_), transferrin saturation (TSAT) in anemic patients, and LDL cholesterol (LDL-C), blood hemoglobin, and urine panels (urine albumin/gram creatinine or urine protein/gram creatinine). For each, we compared the fraction of follow-up tests obtained within the guideline-recommended intervals (Table 1) and the fraction of values within guideline target ranges (Table 1) for CDSS and control patients. These were our primary endpoints.

**Table 1:** Lab measurement frequency and range targets used in the analysis

Lab measurement	Frequency	Target range
eGFR		
-Stage 3	Every 12 months	
-Stage 4	Every 3 months	
-Stage 5	Every month	
PTH		
- Stage 3		35 pg/mL to 70 pg/mL
- Previous within target	Every 12 months	
- Previous outside target	Every 3 months	
- Stage 4	Every 3 months	70 pg/mL to 110 pg/mL
- Stage 5	Every 3 months	150 pg/mL to 300 pg/mL
Hemoglobin		>10 g/dL
- Females		
- Previous within 12–17	Every 12 months	
- Previous outside 12–17	Every 3 months	
- Males		
- Previous within 13.5–17	Every 12 months	
- Previous outside 13.5–17	Every 3 months	
TSAT (hemoglobin below gender specific range)		>20 %
- Previous within target	Every 12 months	
- Previous outside target	Every 3 months	
25-hydroxyvitamin D		> = 30 ng/mL
- Previous within target	Every 12 months	
- Previous outside target	Every 6 months	
Serum Ca		8.6 to 10.2 mg/dL
- Stage 3		
- Previous within target	Every 12 months	
- Previous outside target	Every 3 months	
- Stage 4	Every 3 months	
- Stage 5	Every month	
Serum Phos		2.7 mg/dL to 4.6 mg/dL
- Stage 3		
- Previous within target	Every 12 months	
- Previous outside target	Every 3 months	
- Stage 4	Every 3 months	
- Stage 5	Every month	
LDL Cholesterol		<100 mg/dL
- Previous within target	Every 12 months	
- Previous outside target	Every 3 months	
CO_2_		22 to 30 mmol/L
- Stage 3		
- Previous within target	Every 12 months	
- Previous outside target	Every 3 months	
- Stage 4	Every 3 months	
- Stage 5	Every month	
Urine Albumin/Creatinine^a^		0 to 29 mg/g
- Previous within target	Every 12 months	
- Previous outside target	Every 3 months	
Urine Protein/Creatinine^a^		0 to 200 mg/g
- Previous within target	Every 12 months	
- Previous outside target	Every 3 months	

^a^Measurement of urine albumin/creatinine or urine protein/creatinine ratio satisfies frequency of urine panel

### Criteria for inclusion in the testing frequency analysis

For each analyte, patient inclusion criteria in the testing interval analysis were based on the adequacy of the ‘follow-up interval’, defined from the first accession that included an eGFR to the last accession available for that patient, plus a three month window. The ‘testing interval’ was defined from the first accession to the next test of that specific analyte. If either the testing interval or the ‘recommended interval’ (Table 1) was less than the follow-up interval, the patient was included in the analysis. After a patient’s last observed accession, we could not know if that patient would ever be tested again at LabCorp. As such we assumed patients were still ‘LabCorp patients’ for up to 3 months following their last known accession date. We performed a sensitivity analysis of the ordering results for selected lab tests, using a 1 month and 6 month assumption on the dropout. There was no qualitative difference in the results when compared to the 3 month assumption. Therefore only the 3 month results are presented.

In some cases, analytes were not available at the start of the follow-up interval. For example, PTH may not have been measured, even though an eGFR result was available. As a study convention, we assumed the result (PTH, for example) was within the target range for the CKD stage; we compared timing to the next measurement from the entry point to guideline recommendations assuming a value within the target range at entry (Table 1).

### Criteria for success for the testing frequency analysis

If the testing interval was less than or equal to the recommended interval, we considered the outcome a success. If the testing interval exceeded the recommended interval, we considered the outcome a failure. In other words, there was no penalty for over-testing, only under-testing. We allowed a grace window of 14 days and 30 days for recommended testing intervals of 3 months and 12 months, respectively.

### Criteria for inclusion and success for guideline target analysis

For the assessment of achieving guideline target lab values, every relevant analyte in every available accession was included for each patient. A binary indicator was created for each lab result. If the lab value fell within the recommended target range, the observation was considered a success. Otherwise, the observation was considered a failure.

### Statistical analysis

Logistic regression was used to compare the odds of ordering compliance between CDSS and control patients. Although patients were sometimes followed for multiple intervals, the current analysis focused on the first recommended accession following the baseline visit. This decision was based on the fact that many patients were not “at risk” for a second accession and because the study sampling scheme makes it impossible to determine if an absence without accessions is due to patient dropout. Each patient received a binary indicator of compliance as described above. Comparisons of the odds of compliance between CDSS and control patients were adjusted for age at entry into the cohort, gender, CKD stage, and physician specialty using logistic regression. Parameter estimates were obtained via maximum likelihood, and corresponding confidence intervals and *p*-values were computed using a normal approximation. To test the hypothesis that the effect of CDSS involvement on ordering compliance differs by CKD stage and/or physician specialty, multiplicative interactions between CDSS and CKD stage/physician specialty were tested using the likelihood ratio test. Analytic results are presented using all available patients and are stratified by CKD stage and physician specialty. Analyses accounting for possible correlation within physician revealed no qualitative change in inference.

Generalized estimating equations with a logit link function were used to compare the odds of target compliance between CDSS and control practices. Because a patient could contribute multiple observations (one for each lab accession), we accounted for within-subject correlation across measurements using working correlation structures which were chosen using empirical covariance matrices. Robust variance estimates clustering on patient were used for inference regarding model parameters [24]. All analyses were adjusted for age at entry into the cohort, gender, CKD stage, and physician specialty. Again, interactions by CDSS status and CKD stage and physician specialty were considered.

## Results

CDSS patients and controls were well balanced across the majority of baseline characteristics due to the matched design (Table 2). With one exception, tests of differences between the two groups were significant at the 0.05 level simply because of the large sample size available rather than because of clinically meaningful differences between the groups. The exception was that CDSS patients were cared for by a slightly higher proportion of nephrologists (34.0 vs. 27.4 %) and less primary care physicians (PCPs) (63.9 vs. 70.2 %) when compared to control patients.

**Table 2:** Baseline patient characteristics

Baseline characteristic	Controls (*N* = 42,996)	CDSS patients (*N* = 12,353)	*p*-value
Baseline Age (yrs)	76.09 (11.51)	73.03 (11.51)	<.001
Gender			
- Female	24673 (57.4 %)	7074 (57.3 %)	0.822
- Male	18323 (42.6 %)	5279 (42.7 %)	
Race			
- White-Caucasian	1628 (3.8 %)	491 (4 %)	0.865
- African-American	799 (1.9 %)	242 (2 %)	
- Asian	35 (0.1 %)	11 (0.1 %)	
- Hispanic	41 (0.1 %)	12 (0.1 %)	
- Other	16 (0 %)	6 (0 %)	
- Missing	40477 (94.1 %)	11591 (93.8 %)	
Baseline eGFR	41.95 (11.36)	41.51 (11.41)	<.001
- 15–29	7198 (16.7 %)	2195 (17.9 %)	
- 30–44	15883 (36.9 %)	4559 (37.1 %)	
- 45–59	19915 (46.3 %)	5525 (45 %)	
Physician Specialty			
- Primary Care	30173 (70.2 %)	7896 (63.9 %)	<.001
- Nephrology	11802 (27.4 %)	4198 (34 %)	
- Other	1021 (2.4 %)	259 (2.1 %)	

For Baseline Age and Baseline eGFR, numbers are means (standard deviations). For all other characteristics, numbers are N (%)

Physicians who used the CDSS ordered all CKD relevant testing more in accord with guidelines than those who did not use the system (Table 3). The increase in odds for CDSS relative to non-CDSS ranged from 29 % for TSAT to 88 % for phosphorus. Of interest, the absolute success rates for timely testing were generally not very high (less than 50 %), except for eGFR, calcium, CO_2_, and hemoglobin (Table 3).

**Table 3:** Estimated odds ratio for ordering laboratory tests within guideline-recommended intervals: CDSS vs Controls

Test	Group	N	N (%) in compliance	OR (CI)^a^	*p*-value
eGFR	CDSS	7518	6664 (89)	1.59 (1.45, 1.74)	<0.001
	Control	18979	15462 (81)		
PTH	CDSS	8030	2106 (26)	1.68 (1.57, 1.80)	<0.001
	Control	20121	3405 (17)		
25-D	CDSS	7630	3638 (48)	1.60 (1.51, 1.69)	<0.001
	Control	17132	5942 (35)		
Calcium	CDSS	8335	7076 (85)	1.65 (1.54, 1.77)	<0.001
	Control	20718	15683 (76)		
Phosphorus	CDSS	7653	3096 (41)	1.88 (1.76, 2.01)	<0.001
	Control	19247	5129 (27)		
CO2	CDSS	9173	7475 (82)	1.87 (1.75, 1.98)	<0.001
	Control	23089	16158 (70)		
Hemoglobin	CDSS	9290	6163 (66)	1.82 (1.73, 1.92)	<0.001
	Control	22500	11593 (52)		
TSAT	CDSS	9270	1321 (14)	1.29 (1.20, 1.39)	<0.001
	Control	22414	2540 (11)		
LDL-C	CDSS	8017	4488 (56)	1.79 (1.70, 1.89)	<0.001
	Control	18640	7776 (42)		
Urine panel	CDSS	6985	2265 (32)	1.56 (1.46, 1.67)	<0.001
	Control	15874	3525 (22)		

^a^Values are odds ratios for ordering CKD relevant testing in accordance with guidelines after adjustment for age at entry into the cohort, gender, CKD stage, and physician specialty

**Fig. 1:**
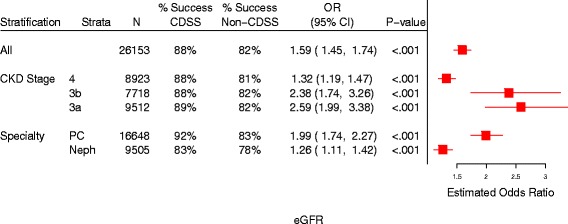
Effect of CDSS on adherence to guideline testing intervals for eGFR. ‘% Success’ refers to percentage of patients with test ordered within the guideline-recommended time interval. Odds ratios greater than one favor CDSS. PC = primary care, Neph = nephrology, N = number of patients, OR = odds ratio

**Fig. 2:**
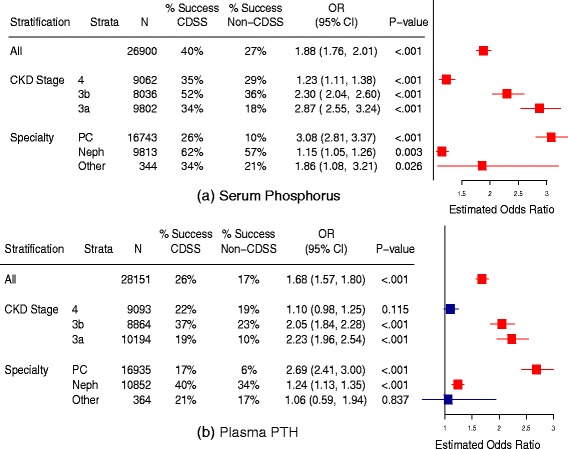
Effect of CDSS on adherence to guideline testing intervals for phosphorus (**a**) and PTH (**b**). ‘% Success’ refers to percentage of patients with test ordered within the guideline-recommended time interval. Odds ratios greater than one favor CDSS. PC = primary care, Neph = nephrology, N = number of patients, OR = odds ratio

**Fig. 3:**
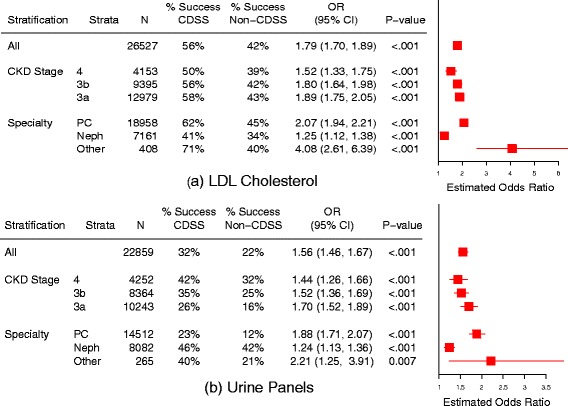
Effect of CDSS on adherence to guideline testing intervals for LDL-C (**a**) and Urine Panels (**b**). ‘% Success’ refers to percentage of patients with test ordered within the guideline-recommended time interval. Odds ratios greater than one favor CDSS. The interaction between CDSS and both CKD stage and physician type was significant (*p* < 0.001), meaning that the benefits conferred by CDSS varied with CKD stage and physician type. PC = primary care, Neph = nephrology, N = number of patients, OR = odds ratio

The magnitude of the benefit conferred by CDSS was greater for PCPs than nephrologists for all of the tests we studied; eGFR, phosphorus, PTH, LDL-C, and urine panels are illustrated in Figs. 1, 2 and 3. The CDSS effect was greater among stage 3 compared to stage 4 patients for eGFR, phosphorus, and PTH (Figs. 1 and 2), as well as for calcium, CO_2_, and TSAT (not illustrated).

**Fig. 4:**
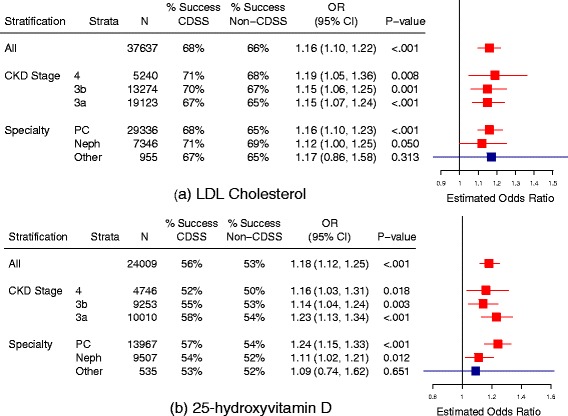
Effect of CDSS on achievement of guideline targets for LDL cholesterol (**a**) and 25-D (**b**). ‘% Success’ refers to percentage of patients whose test result met the guideline recommended target. Odds ratios greater than one favor CDSS. PC = primary care, Neph = nephrology, N = number of patients, OR = odds ratio

CDSS physicians met guideline targets for LDL-C and 25-D more often than control physicians (Fig. 4). The CDSS effect did not differ by CKD stage or physician specialty (Fig. 4). By contrast, CDSS physicians met hemoglobin targets less often than control physicians (Table 4). CDSS did not affect the achievement of the remaining guideline targets (Table 4).

**Table 4:** Estimated odds ratio for achieving guideline-recommended laboratory test targets: CDSS vs Controls

Test	Group	N	N (%) in compliance	OR (CI)^a^	*p*-value
PTH	CDSS	12835	4918 (38)	0.99 (0.93, 1.06)	0.851
	Control	23582	9062 (38)		
25-D	CDSS	16910	9332 (55)	1.18 (1.12, 1.25)	<0.001
	Control	32903	17362 (53)		
Calcium	CDSS	67222	59847 (89)	1.05 (0.99, 1.10)	0.113
	Control	167026	148877 (89)		
Phosphorus	CDSS	17764	15456 (87)	1.08 (0.99, 1.06)	0.069
	Control	29011	24605 (85)		
CO2	CDSS	167237	120910 (72)	0.99 (0.96, 1.03)	0.657
	Control	68208	47701 (70)		
Hemoglobin	CDSS	47740	18528 (39)	0.92 (0.88, 0.97)	0.001
	Control	45229	108476 (42)		
TSAT	CDSS	8119	5495 (68)	0.98 (0.90, 1.07)	0.699
	Control	15717	10716 (68)		
LDL-C	CDSS	25278	17232 (68)	1.16 (1.10, 1.22)	<0.001
	Control	63738	41957 (66)		
Urine panel	CDSS	8806	4263 (48)	0.94 (0.87, 1.02)	0.156
	Control	25814	12877 (50)		

^a^Values are estimated odds ratios for meeting guideline target values of CKD relevant laboratory measurements after adjustment for age at entry into the cohort, gender, CKD stage, and physician specialty

## Discussion

### Guidelines for testing frequency

Compared to non-CDSS physicians, CDSS physicians adhered better to guidelines that concern timely ordering of CKD laboratory testing. To the extent that the guidelines are themselves valid and useful for betterment of patient care, these results speak to a corresponding benefit of the CDSS. Being merely an addition to an otherwise conventional laboratory report, our CDSS enters practices through conventional workflow portals, imposes no specific burdens of its own, and is scalable to whatever population size one desires to reach.

Our data underscore the importance of improving CKD care because, even with CDSS, no more than half of laboratory orders were consistent with guideline recommendations, except for eGFR, calcium, CO_2_, and hemoglobin. Three of these four exceptions, eGFR, calcium, and CO_2_, are part of the most widely ordered test panels. The low accordance with guidelines (under-testing) for the remaining tests was true even among nephrologists. We did not penalize for over-testing because many of these tests are routinely ordered for clinical indications outside of CKD monitoring and we had no means to discern this.

We assumed that missing values in the initial accession were within the target range for the CKD stage. This assumption gave all physicians the maximum interval to the next order and, therefore, imposed the least stringency in selection of the proper time for re-testing. For example, if the initial accession for a stage 3 CKD patient did not include a PTH, we assumed the PTH was within goal and allowed a testing interval of one year versus the shorter interval– 3 months –recommended for an abnormal PTH. This greater leniency unselectively advantaged all physicians and diluted the CDSS effect.

As expected, the benefits of CDSS were more marked for PCPs than nephrologists. The latter have special training and experience in CKD management, have been the target of guideline material through journals and scientific meetings, and would be expected to need less help than PCPs in managing CKD patients. The effect among nephrologists validates the idea that the program can improve the performance of even specialists.

### Guidelines for laboratory targets

CDSS physicians achieved better laboratory outcomes for two measurements: LDL-C and 25-D. Our CDSS aims toward lower LDL-C levels than usual because CKD is a very high risk state for cardiovascular disease (CVD) [25]. Likewise, 25-D deficiency is common in CKD and a major contributor to secondary hyperparathyroidism [26, 27]. Advice in these areas for more aggressive LDL-C lowering and 25-D repletion may have been a stimulus to more intensive medical care. The benefit of CDSS was similar across CKD stage and for all physician specialties. Of note, the KDOQI intact PTH targets used in this analysis are opinion-based [12] and no longer endorsed by more recent KDIGO guidelines [17]; this could have contributed to the lack of achievement of PTH targets in this study. Since completion of this study, the PTH goals promulgated in this CDSS have been updated to reflect the KDIGO guidelines. It may be that future CDSS studies using more widely accepted guidelines would demonstrate a higher rate of compliance.

### Strengths and limitations of the study

There are several strengths to this study, such as the large sample size, the wide geographic distribution, and the range of practice sizes and specialties. It is true that, as this was not a randomized controlled trial (RCT), timely ordering and better achievement of targets could be attributed to chance association. On the other hand, an RCT concerning physician behavior imposes stringencies that limit generalizability. Physicians who volunteer to participate in an RCT may not represent the majority of physicians in practice. The uniform records and research coordinators required by RCTs distort the process of clinical care. Our study occurred during the natural course of medical practice by the very physicians who are the target audience of the guidelines: those caring for CKD patients.

The brevity of this study (median follow up 0.73 years) and its essential design permit no assessment of what impact the CDSS program might have on crucial issues such as CKD progression. If the guidelines are helpful in reducing the rate of GFR decline, and if our program is likewise effective in implementing them over the time intervals needed to assess CKD progression, then our program might be a means for maintaining kidney function. These uncertainties point to the need for a longer study. Additionally, ethnicity was not known for most of our patients, so matching was imperfect in this respect. The default use of the non-African American eGFR calculation when race was unknown would tend to underestimate GFR and consequently over-diagnose CKD in African American patients. Because the CDSS did not ‘know’ the correct eGFR to use and physicians did know, the performance of CDSS in relation to guidelines would be reduced compared to control physicians whenever eGFR was critical in the decision making.

Due to the way in which the program was proliferated, we were unable to determine what proportion of providers were approached, agreed to receive the CDSS, or refused the program. Likewise, we cannot provide information about provider characteristics such as age years since training, size of practices, or LabCorp market share in the given practice locale. We also lack data concerning comorbidities, socioeconomic status and insurance status for the patients. However provider zip code was used as a surrogate for socioeconomic status. In addition, because patients were in various stages of CKD and therefore varied in testing recommendations, each analyte analysis represents that subset of patients due for testing. Finally, certain unavoidable biases are inevitable. Patients with elevated values are likely to have more frequent rechecks than those with goal values. Patients with multiple results may well have more complex and therapeutically recalcitrant disease. The potential use of outside labs by a provider may have occurred but seems equally likely in either group.

The providers themselves are surely a source of selection bias. Those more interested may well have chosen to use CDSS. On the other hand, many of the physicians in the control group were not made aware of our CDSS program by the LabCorp sales force. This problem cannot be resolved within the present data set. We cannot assess provider performance in CKD care prior to our study because LabCorp did not keep a patient-centered database at that time.

### Prior studies of CDSS

#### CKD CDSS

Manns et al. studied 93 primary care practices in Canada treating 22,092 patients with diabetes or proteinuria [21]. Practices were randomized to receive either an enhanced eGFR prompt containing suggestions about ACEi or ARB use, blood pressure control, lipid control, and A1c target, or a standard eGFR prompt. Although the primary outcome, which concerned use of ACEi/ARB, was limited to the 5444 patients over age 65 for whom data were available, the results that concern laboratory testing were based upon all patients. They found the prompt improved the rate of urine albumin measurements, but not that of LDL-C or HbA1c measurements. The enhanced prompt did not alter the achievement of laboratory test targets.

In another study [20], real-time automated alerts recommending nephrology referral and urine albumin measurements failed to significantly improve either endpoint. Studies of CDSS aimed at reducing adverse drug events or renal function impairment in CKD patients have shown benefits only in reducing medication errors [28, 29].

All in all, the record of CDSS usage for CKD has not been such as to promote its widespread adoption. By contrast, our CDSS might have practical value. It is scalable, and imposes no extra effort on physicians or their support staff. Our CDSS is specific to an individual patient and cognizant of changes over time. At the very least, it appears to improve the timeliness of lab test ordering. It may, in some cases, lead to better achievement of laboratory targets.

#### Non-CKD CDSS

Our results are in line with or perhaps more promising than those from non-CKD trials of CDSS. Bright et al. reviewed 148 RCTs of CDSS [9]. Of these, 128 were health care process trials like ours. Overall, CDSS improved ordering of clinical studies that included laboratory testing and procedures, preventative care services, and treatments. The overall odds ratio averaged 1.72, similar to the present study. CDSS tended to improve morbidity outcomes such as hospitalizations, cardiovascular events, DVTs, and infections. Mortality was not affected. Studies of cost-effectiveness were not consistent. All in all, the general results for this meta-analysis are in reasonable accord with our own results, even though ours was not an RCT. Only 20 of the studies in this meta-analysis dealt with lab test ordering. Almost none were national in scope like ours; most were performed at one or a few sites, except for a large study at Kaiser Permanente [30].

Three studies published subsequent to Bright et al. found somewhat more negative results. A meta-analysis of 15 diabetes management trials concluded that CDSS did not improve practitioner performance or patient outcomes [5]. Eaton et al. performed a cluster RCT to evaluate a CDSS for improving adherence to cholesterol guidelines in 30 New England primary care practices and found no significant differences in lipid screening or achievement of cholesterol goals [6]. Anchala et al. evaluated ten RCTs and observational studies of CDSS in blood pressure management and CVD prevention [31]. Pooled results for reduction of systolic blood pressure were not significant. Although individual studies suggested modest improvements in outcomes such as reductions in acute myocardial infarction and cardiovascular re-hospitalization, data were too sparse to demonstrate a definitive benefit of CDSS for CVD prevention.

Roshanov et al. reviewed 162 RCTs concerning what factors associated with success or failure of CDSS [32]. They found that 58 % of RCTs showed some benefit of clinical process or patient outcomes. CDSS that provided advice for patients as well as physicians, required a reason for overriding the CDSS, or were tested by the CDSS creators showed better results. Systems that presented advice within electronic health records or order entry systems were less likely to be effective. Our CDSS possesses several of the characteristics associated with success: providing advice for patients and physicians, testing by the creators, and delivery outside the electronic health record and ordering system.

## Conclusions

Our study illustrates that a national laboratory is a platform of sufficient scalability to deliver CDSS programs to physicians throughout the country via their natural workflow portals and thereby transmit guidelines to their intended audiences. This CDSS seems a promising tool to improve guideline-based laboratory test ordering and perhaps outcomes in CKD. We believe it is the first to be reported by a national laboratory. That large scale systems can deliver guidelines into practices, and thereby affect the behavior of large numbers of physicians, means that guideline authors may well acquire increasing influence and a correspondingly greater responsibility.

## Additional files

Additional file 1.
**List of Scientific Advisory Board Members.** (DOCX 16 kb) Additional file 2.
**Sample guideline-based report to physicians.** (PDF 111 kb) Additional file 3.
**Sample flow sheets of relevant test results.** (PDF 82 kb) Additional file 4.
**Sample graph page of relevant test results.** (PDF 289 kb) Additional file 5.
**Sample patient educational materials.** (PDF 97 kb) Additional file 6.
**Sample dietary educational brochure.** (PDF 4781 kb)

## References

[1] Cabana MD, Rand CS, Powe NR, Wu AW, Wilson MH, Abboud PA, Rubin HR (1999). Why don’t physicians follow clinical practice guidelines? A framework for improvement. JAMA.

[2] Davis DA, Taylor-Vaisey A (1997). Translating guidelines into practice. A systematic review of theoretic concepts, practical experience and research evidence in the adoption of clinical practice guidelines. CMAJ.

[3] Patwardhan MB, Samsa GP, Matchar DB, Haley WE (2007). Advanced chronic kidney disease practice patterns among nephrologists and non-nephrologists: a database analysis. Clin J Am Soc Nephrol.

[4] Jones JB, Stewart WF, Darer JD, Sittig DF (2013). Beyond the threshold: real-time use of evidence in practice. BMC Med Inform Decis Mak.

[5] Jeffery R, Iserman E, Haynes RB (2013). Can computerized clinical decision support systems improve diabetes management? A systematic review and meta-analysis. Diabet Med.

[6] Eaton CB, Parker DR, Borkan J, McMurray J, Roberts MB, Lu B, Goldman R, Ahern DK (2011). Translating cholesterol guidelines into primary care practice: a multimodal cluster randomized trial. Ann Fam Med.

[7] Schnipper JL, Linder JA, Palchuk MB, Yu DT, McColgan KE, Volk LA, Tsurikova MA, Melnikas BA, Einbinder JS, Middleton B (2010). Effects of documentation-based decision support on chronic disease management. Am J Manag Care.

[8] Gill JM, Chen YX, Glutting JJ, Diamond JJ, Lieberman MI (2009). Impact of decision support in electronic medical records on lipid management in primary care. Popul Health Manag.

[9] Bright TJ, Wong A, Dhurjati R, Bristow E, Bastian L, Coeytaux RR, Samsa G, Hasselblad V, Williams JW, Musty MD, Wing L, Kendrick AS, Sanders GD, Lobach D (2012). Effect of clinical decision-support systems: a systematic review. Ann Intern Med.

[10] National Kidney Foundation (2002). K/DOQI clinical practice guidelines for chronic kidney disease: evaluation, classification, and stratification. Am J Kidney Dis.

[11] National Kidney Foundation (2003). K/DOQI clinical practice guidelines for management of dyslipidemias in patients with kidney disease. Am J Kidney Dis.

[12] National Kidney Foundation (2003). K/DOQI clinical practice guidelines for bone metabolism and disease in chronic kidney disease. Am J Kidney Dis.

[13] National Kidney Foundation (2006). KDOQI Clinical Practice Guidelines and Clinical Practice Recommendations for Anemia in Chronic Kidney Disease. Am J Kidney Dis.

[14] National Kidney Foundation (2004). K/DOQI clinical practice guidelines on hypertension and antihypertensive agents in chronic kidney disease. Am J Kidney Dis.

[15] Kidney Disease: Improving Global Outcomes (KDIGO) Anemia Work Group (2012). KDIGO clinical practice guideline for anemia in chronic kidney disease. Kidney Int Suppl.

[16] Kidney Disease: Improving Global Outcomes (KDIGO) CKD Work Group (2013). KDIGO 2012 Clinical Practice Guideline for the Evaluation and Management of Chronic Kidney Disease. Kidney Int Suppl.

[17] Kidney Disease: Improving Global Outcomes (KDIGO) Blood Pressure Work Group (2009). KDIGO clinical practice guideline for the diagnosis, evaluation, prevention, and treatment of Chronic Kidney Disease-Mineral and Bone Disorder (CKD-MBD). Kidney Int Suppl.

[18] Kidney Disease: Improving Global Outcomes (KDIGO) Blood Pressure Work Group (2012). KDIGO clinical practice guideline for the management of blood pressure in chronic kidney disease. Kidney Int Suppl.

[19] Kidney Disease: Improving Global Outcomes (KDIGO) Lipid Work Group (2013). KDIGO clinical practice guideline for lipid management in chronic kidney disease. Kidney Int Suppl.

[20] Abdel-Kader K, Fischer GS, Li J, Moore CG, Hess R, Unruh ML (2011). Automated clinical reminders for primary care providers in the care of CKD: a small cluster-randomized controlled trial. Am J Kidney Dis.

[21] Manns B, Tonelli M, Culleton B, Faris P, McLaughlin K, Chin R, Gooch K, McAlister FA, Taub K, Thorlacius L, Krause R, Kearns M, Hemmelgarn B (2012). A cluster randomized trial of an enhanced eGFR prompt in chronic kidney disease. Clin J Am Soc Nephrol.

[22] Fox CH, Vest BM, Kahn LS, Dickinson LM, Fang H, Pace W, Kimminau K, Vassalotti J, Loskutova N, Peterson K (2013). Improving evidence-based primary care for chronic kidney disease: study protocol for a cluster randomized control trial for translating evidence into practice (TRANSLATE CKD). Implement Sci.

[23] Wright NJ, Greene JH, Wallston K, Eden S, Shintani A, Elasy T, Rothman RL, Ikizler TA, Cavanaugh KL (2013). Pilot study of a physician-delivered education tool to increase patient knowledge about CKD. Am J Kidney Dis.

[24] Zeger SL, Liang KY (1986). Longitudinal data analysis for discrete and continuous outcomes. Biometrics.

[25] Briasoulis A, Bakris GL (2013). Chronic kidney disease as a coronary artery disease risk equivalent. Curr Cardiol Rep.

[26] LaClair RE, Hellman RN, Karp SL, Kraus M, Ofner S, Li Q, Graves KL, Moe SM (2005). Prevalence of calcidiol deficiency in CKD: a cross-sectional study across latitudes in the United States. Am J Kidney Dis.

[27] Levin A, Bakris GL, Molitch M, Smulders M, Tian J, Williams LA, Andress DL (2007). Prevalence of abnormal serum vitamin D, PTH, calcium, and phosphorus in patients with chronic kidney disease: results of the study to evaluate early kidney disease. Kidney Int.

[28] Chang J, Ronco C, Rosner MH (2011). Computerized decision support systems: improving patient safety in nephrology. Nat Rev Nephrol.

[29] Milani RV, Oleck SA, Lavie CJ (2011). Medication errors in patients with severe chronic kidney disease and acute coronary syndrome: the impact of computer-assisted decision support. Mayo Clin Proc.

[30] Palen TE, Raebel M, Lyons E, Magid DM (2006). Evaluation of laboratory monitoring alerts within a computerized physician order entry system for medication orders. Am J Manag Care.

[31] Anchala R, Pinto MP, Shroufi A, Chowdhury R, Sanderson J, Johnson L, Blanco P, Prabhakaran D, Franco OH (2012). The role of Decision Support System (DSS) in prevention of cardiovascular disease: a systematic review and meta-analysis. PLoS One.

[32] Roshanov PS, Fernandes N, Wilczynski JM, Hemens BJ, You JJ, Handler SM, Nieuwlaat R, Souza NM, Beyene J, Van Spall HG, Garg AX, Haynes RB (2013). Features of effective computerised clinical decision support systems: meta-regression of 162 randomised trials. BMJ.

