# Supplementary material for: Clinical decision support improves physician guideline adherence for laboratory monitoring of chronic kidney disease: a matched cohort study
Source: BMC Nephrol. 2015 Oct 15;16:163. doi: 10.1186/s12882-015-0159-5 (PMC4608162; doi:10.1186/s12882-015-0159-5)
Supplement: Additional file 1. — List of Scientific Advisory Board Members. (DOCX 16 kb) [file 12882_2015_159_MOESM1_ESM.docx]

**Scientific Advisory Board Members**

Fredric L. Coe, MD, Chairman

Professor of Medicine, University of Chicago Pritzker School of Medicine, Chicago, Illinois

Stuart Sprague, DO

Professor of Medicine and Chief, Division of Nephrology & Hypertension, Northshore University HealthSystem, University of Chicago Pritzker School of Medicine, Evanston, Illinois

Sharon M. Moe, MD

Professor of Medicine, Nephrology Division Director, Indiana University School of Medicine and Section Chief of Nephrology at Roudebush Veterans Administration Medical Center, Indianapolis, IN

Bertram L. Kasiske, MD

Professor of Medicine and Head of Transplant Nephrology, Director of the Renal Division at Hennepin County Medical Center, University of Minnesota Medical School, Minneapolis, MN

 Elaine M. Worcester, MD

Professor of Medicine, University of Chicago Pritzker School of Medicine, Chicago, Illinois

 Jeffrey Berns, MD

Professor of Medicine, Associate Chief, Renal Division, Director, Renal Fellowship Program, University of Pennsylvania School of Medicine, Philadelphia, PA

Adeera Levin, MD

Professor, Division of Nephrology, University of British Columbia, Executive Director, BC Provincial Renal Agency, Vancouver, BC, Canada

George Bakris, MD

Professor of Medicine, Director, Comprehensive Hypertension Center, University of Chicago Pritzker School of Medicine, Chicago, IL

Robert Toto, MD

Professor of Medicine, Associate Dean of Translational Science, University of Texas Southwestern Medical School, Dallas, TX

Joseph Vassalotti, MD

Chief Medical Officer, National Kidney Foundation, Associate Clinical Professor of Medicine, Icahn School of Medicine at Mount Sinai, New York, NY

Michael Rocklin, MD

Director of Clinical Quality, Denver Nephroloy, Clinical Instructor, University of Colorado Health Sciences Center, Denver, Colorado

Geoffrey Block, MD

Chief of Clinical Research, Denver Nephroplogy, Associate Clinical Professor of Medicine, University of Colorado Health Sciences Center, Denver, Colorado

Daniel L. Gillen, PhD

Associate Professor of Statistics, University of California at Irvine, Irvine, California

Arthur H. Rubenstein, MBBCh

Executive Vice President, University of Pennsylvania Health System, Robert G. Dunlop Professor of Medicine and Dean of the School of Medicine, University of Pennsylvania, Philadelphia, Pennsylvania, Former Director, Laboratory Corporation of America
