# Supplementary material for: Clinical decision support improves physician guideline adherence for laboratory monitoring of chronic kidney disease: a matched cohort study
Source: BMC Nephrol. 2015 Oct 15;16:163. doi: 10.1186/s12882-015-0159-5 (PMC4608162; doi:10.1186/s12882-015-0159-5)
Supplement: Additional file 2. — Sample guideline-based report to physicians. (PDF 111 kb) [file 12882_2015_159_MOESM2_ESM.pdf]

KDOQI™ Based Analysis and Treatment Options

GENDER	DIABETES	SELF-REPORTED RACE	CURRENT eGFR	MOST RECENT CKD STAGE
M	Yes	White-Caucasian	**46**	**3**

eGFR, Blood Pressure, and Proteinuria

The overall regression of eGFR with time is not statistically significant. Date of blood pressure measurement reported by your office is 05/17/09. Systolic Blood Pressure is above goal and has risen, was 135 and now is 150 mm Hg. Diastolic Blood Pressure is above goal and has risen, was 82 and now is 94 mm Hg. Potassium is within goal and has decreased, was 5.2 and now is 4.3 mmol/L. ADA recommends hemoglobin A1C should be obtained every 3 months in a diabetic not meeting glycemic goals. The following treatment information was reported by your office on 05/18/09: IN USE - ACEI or ARB, NOT IN USE - diuretic.

Treatment Suggestions [1]

Confirm adherence to antihypertensive therapy. Consider increase of ACEI or ARB, unless at maximal dose. Consider thiazide diuretic for blood pressure control. Lifestyle should include sodium intake of 2300 to 3000 mg/d, exercise (30 minutes 5 times a week), less than three alcoholic drinks per day, a low dose (81 mg) aspirin daily, and smoking cessation.

Bone & Mineral

Intact PTH is above goal and has not changed significantly, was 82 and now is 89 pg/mL. Phosphorus is within goal and has risen, was 3.2 and now is 4.1 mg/dL. Corrected calcium is within goal and has risen, was 8.7 and now is 9.1 mg/dL. Carbon Dioxide is within goal and has risen, was 17 and now is 25 mmol/L. Vitamin D deficiency is present (was 8 and now is 9 ng/mL). The following treatment information was reported by your office on 05/18/09: NOT IN USE - low phosphate diet, phosphate binders, active vitamin D analog, vitamin D, and alkali.

Treatment Suggestions [1]

Begin vitamin D (ergocalciferol 50,000 IU/week orally for 4 weeks then monthly for 5 months; alternatively, cholecalciferol 2000 IU/d for 6 months). Restrict diet phosphate to 800 - 1000 mg/d. Phosphate binder would be used to lower PTH, if 25-hydroxy vitamin D was in the normal range.

Lipids

LDL is within goal and has decreased, was 133 and now is 55 mg/dL. Triglyceride is within goal and has decreased, was 91 and now is 66 mg/dL. Non-HDL Cholesterol is within goal and has decreased, was 156 and now is 58 mg/dL. HDL is within goal and has not changed significantly, was 67 and now is 67 mg/dL. The following treatment information was reported by your office on 05/18/09: IN USE - statin, NOT IN USE - fibrate and niacin.

Treatment Suggestions [1]

Therapeutic lifestyle changes are always valuable to maintain optimal blood lipid status (diet, exercise, weight management). Continue statin.

Anemia

Hemoglobin is low and has not changed significantly, was 12.2 and now is 12.2 g/dL. TSAT is within goal and has risen, was 13.0 and now is 21.0 %. Ferritin is within goal and has risen, was 23 and now is 51 ng/mL. The following treatment information was reported by your office on 05/18/09: IN USE - iron, NOT IN USE - ESA.

Treatment Suggestions [1]

Continue iron therapy. ESA therapy is not recommended in CKD until hemoglobin is less than 10 g/dL.

Follow-Up Suggestions[1]

Due
- Spot Urine Panel (Albumin preferred)
- Hemoglobin A1C

1 month
- Fasting Renal Panel

3 months
- Fasting PTH with Renal Panel
- Fasting Lipid Panel
- Fe/TIBC (TSAT) and Ferritin with CBC

6 months
- 25-Hydroxy Vitamin D

[1] **Disclaimer**: These treatment and follow-up suggestions are based primarily on the KDOQI™ guidelines, are computer generated, and are subject to error. The treating physician should not rely solely on this guidance. Litholink does not have a complete medical history. See the disclaimer on page 1.

Mitchell S. Laks, PhD
Laboratory Director
CLIA# 14D0897314

Litholink Corporation
2250 West Campbell Park Drive
Chicago, Illinois 60612

800 338 4333 Telephone
866 361 7939 Facsimile
www.litholink.com
