# Supplementary material for: Clinical decision support improves physician guideline adherence for laboratory monitoring of chronic kidney disease: a matched cohort study
Source: BMC Nephrol. 2015 Oct 15;16:163. doi: 10.1186/s12882-015-0159-5 (PMC4608162; doi:10.1186/s12882-015-0159-5)
Supplement: Additional file 3. — Sample flow sheets of relevant test results. (PDF 82 kb) [file 12882_2015_159_MOESM3_ESM.pdf]

PATIENT	DATE OF BIRTH	CKD STAGE	DATE OF SERVICE	PHYSICIAN
**CKD Patient 1, Example**	**XX/XX/XXXX**	**3**	**05/17/2009**	**Physician, Sample**

Test Results & Treatments

The 8 most recent lab results as well as the treatments are reported.

eGFR

Lab Date	K	BUN	SCr	eGFR [2]	HB A1c	Protein/Cr (mg/g)	Alb/Cr (ug/mg)	Weight (kg)	SBP [3]	DBP	Date Meds Reported	ACEI/ARB	Diuretic
05/17/09	**4.3**	**26**	**1.60**	**46**				**72.1**	**150**	**94**	05/18/09	**Yes**	**No**
02/27/09	5.2	34	1.50	50				70.3	135	82	02/28/09	Yes	No
01/06/09	4.7	49	1.60	46	7.8			69.4	?	?	01/07/09	Yes	No
01/03/09	4.4	41	1.50	50				74.4	?	?	01/04/09	Yes	No
01/13/08	4.8	25	1.70	43		176		74.4	135	78	01/14/08	Yes	No

[2] Effective April 4, 2011, Litholink is reporting the eGFR using the CKD-EPI equation. The use of this equation allows for reporting of eGFR values greater than 59. eGFR values previously reported using the MDRD equation will be recalculated using the CKD-EPI equation. The CKD-EPI equation has only been validated for patients aged 18 to 70.
[3] Our interpretation of blood pressure assumes the reported measurements are representative of the patient's average blood pressure at this time.

Bone & Mineral

Date	PTH	P	Corr Ca	CO_2	K	25-D	LPD	PB	CaPB	AVD	Vit D	Alkali
05/17/09	**89**	**4.1**	**9.1**	**25**	**4.3**	**9.0**	**No**	**No**	**No**	**No**	**No**	**No**
02/27/09		3.2	8.7	17	5.2		No	No	No	No	No	No
01/06/09		3.4	8.5	23	4.7		No	No	No	No	No	No
01/03/09	82	3.4	8.9	17	4.4	8.0	No	No	No	No	No	No
01/13/08	29	4.0	9.4	25	4.8		No	No	No	No	No	No

Lipids

Date	LDL	TG	non-HDL	Chol	HDL	Statin	Fibrate	Niacin	Fasting
05/17/09	**55**	**66**	**58**	**125**	**67**	**Yes**	**No**	**No**	**?**
01/06/09	133	91	156	210	67	Yes	No	No	?
02/10/08	135	91	153	216	63	No	No	No	?

Anemia

Date	Hb	TSAT	Ferritin	Iron	ESA
05/17/09	**12.2**	**21.0**	**51**	**Yes**	**No**
02/27/09	12.2			Yes	No
01/03/09	10.8			Yes	No
02/10/08	12.1	13.0	23	Yes	No
01/13/08	12.1			Yes	No
