# Supplementary material for: Clinical decision support improves physician guideline adherence for laboratory monitoring of chronic kidney disease: a matched cohort study
Source: BMC Nephrol. 2015 Oct 15;16:163. doi: 10.1186/s12882-015-0159-5 (PMC4608162; doi:10.1186/s12882-015-0159-5)

Graphs

eGFR

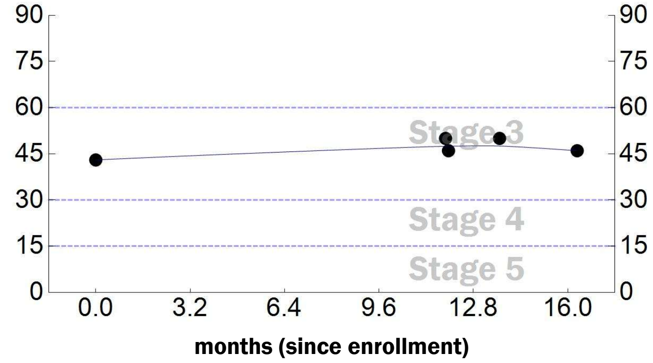

Blood Pressure

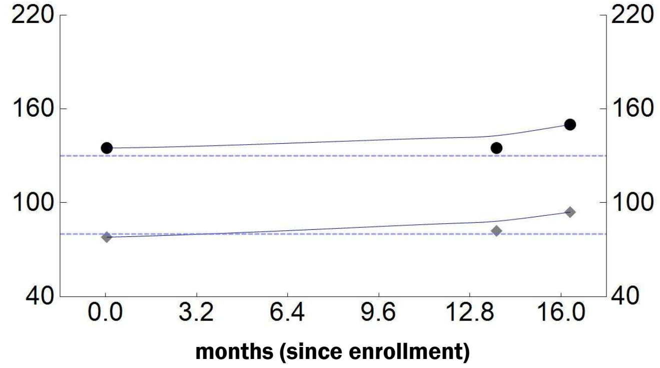

● = plotted based on date measured
◉ = plotted based on date reported (date measured unknown)

PTH

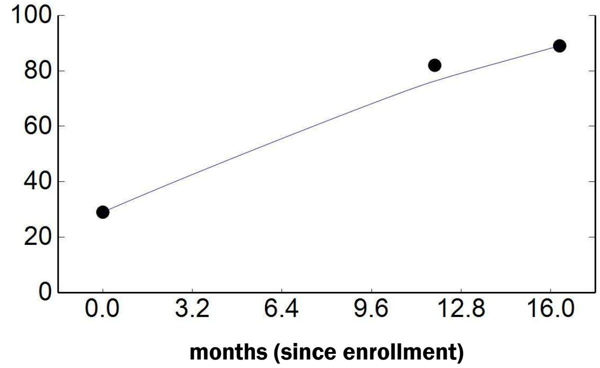

Corrected Calcium

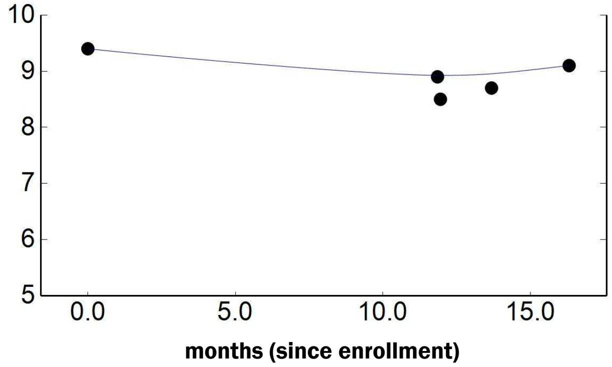

Hemoglobin

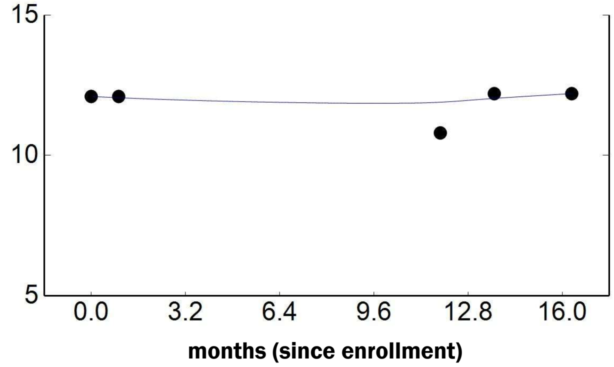

Phosphorus

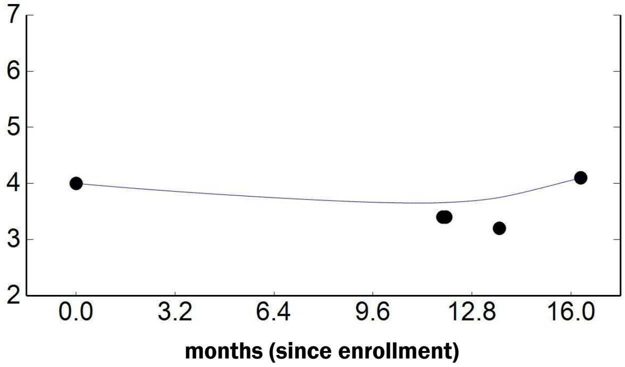

Supplement: Additional file 4. — Sample graph page of relevant test results. (PDF 289 kb) [file 12882_2015_159_MOESM4_ESM.pdf]
