# Supplementary material for: Clinical decision support improves physician guideline adherence for laboratory monitoring of chronic kidney disease: a matched cohort study
Source: BMC Nephrol. 2015 Oct 15;16:163. doi: 10.1186/s12882-015-0159-5 (PMC4608162; doi:10.1186/s12882-015-0159-5)
Supplement: Additional file 5. — Sample patient educational materials. (PDF 97 kb) [file 12882_2015_159_MOESM5_ESM.pdf]

PATIENT	DATE OF BIRTH	CKD STAGE	DATE OF SERVICE	PHYSICIAN
**CKD Patient 1, Example**	**XX/XX/XXXX**	**3**	**05/17/2009**	**Physician, Sample**

Your Test Results

✓ = At Goal

Test	Your Results	Suggestions
**eGFR** estimates how well your kidneys are filtering blood. The higher the number, the better your kidneys are working.	**46**	**YOU ARE IN STAGE 3 CHRONIC KIDNEY DISEASE (CKD).** There are 5 stages of CKD. See below for more information.
**BLOOD PRESSURE (BP):** High BP can damage the heart and kidneys, and may lead to stroke, vision loss, and other problems.	**150/94** (05/17/09) Goal <=130/80	**YOUR BP IS HIGH.** Remember to take all medicines prescribed by your doctor. Avoid foods high in sodium – try to eat 2300 to 3000 mg/day or less. For more information, visit our website or obtain our low sodium diet brochure from your doctor's office. Exercise (at least 30 minutes 5 times a week), weight loss, and quitting smoking can also help to improve BP.
**POTASSIUM** helps keep your heart and muscles working properly. High or low levels can be dangerous.	**4.3** ✓ Goal 3.5-5.2	**YOUR POTASSIUM IS AT GOAL.** To keep it at goal, make sure you avoid foods high in potassium. For more information, visit our website or obtain our low potassium diet brochure from your doctor's office.
**PHOSPHORUS** works with calcium to build strong, healthy bones and keep the rest of the body healthy. Too much phosphorus can weaken bones, and can also damage the heart and blood vessels.	**4.1** ✓ Goal 2.7-4.6	**YOUR PHOSPHORUS IS AT GOAL.** To keep it at goal, continue to avoid foods high in phosphorus. For more information, visit our website or obtain our low phosphorus diet brochure from your doctor's office. Also remember to take your medicines as prescribed by your doctor.
**LDL CHOLESTEROL** is the bad cholesterol and can clog your arteries.	**55** ✓ Goal <=100	**YOUR LDL CHOLESTEROL IS AT GOAL.** To keep it at goal, remember to take any medications prescribed by your doctor. Weight loss, exercise (at least 30 minutes 5 times a week), a low fat diet, and quitting smoking can keep cholesterol low.
**HEMOGLOBIN:** Low hemoglobin is a sign of anemia, or low blood count. It may cause you to feel tired or have low energy.	**12.2** Goal 13.5-17.0	**YOUR HEMOGLOBIN IS LOW.** Low hemoglobin has many causes, and your doctor may order other blood tests to determine the cause. Your doctor may prescribe iron or other treatments if your hemoglobin gets too low. Take all medications as prescribed by your doctor.

YOUR eGFR IS 46, YOU ARE IN STAGE 3 CKD. THINGS YOU NEED TO KNOW:

- CKD means your kidneys have been damaged by diabetes, high blood pressure, or many other diseases. CKD can lead to heart disease.
- Even if you feel fine, you may be at risk for problems such as high blood pressure, high cholesterol, anemia, and bone disease. Your doctor will test you for these problems.
- Avoid taking NSAIDs (e.g. ibuprofen, Advil®, Motrin®, Aleve®) as these may worsen your kidney function.
- To learn more, visit our website or the National Kidney Foundation's website: www.kidney.org/kidneydisease.

For more information on CKD Patient Education, visit www.litholink.com/en/CKDEducation.

Disclaimer: You should discuss this information with your physician. Litholink does not have a doctor-patient relationship with you, nor does it have access to a complete medical history or a physical examination that would be necessary for a complete diagnosis and comprehensive treatment plan. Neither you nor your physician should rely solely on this guidance. The medical bases for the suggestions are the guidelines created by the National Kidney Foundation Kidney Disease Outcomes Quality Initiative (KDOQI™) at www.Kidney.org/professionals/KDOQI. Adapted from: http://nkdep.nih.gov/resources/nkdep-kidney-test-results-508.pdf
