# Supplementary material for: Clinical decision support improves physician guideline adherence for laboratory monitoring of chronic kidney disease: a matched cohort study
Source: BMC Nephrol. 2015 Oct 15;16:163. doi: 10.1186/s12882-015-0159-5 (PMC4608162; doi:10.1186/s12882-015-0159-5)
Supplement: Additional file 6. — Sample dietary educational brochure. (PDF 4781 kb) [file 12882_2015_159_MOESM6_ESM.pdf]

Read food labels

to find the best choice for your diet

Serving size tells you what a single portion is.

Nutrition Facts

Serving Size 6 crackers (28g)
Servings Per Container About 10

Amount Per Serving	
**Calories** 120 Calories from Fat 40	
	**% Daily Value***
**Total Fat** 4.5g	**7**%
Saturated Fat 1g	**5**%
Trans Fat 0g	
Polyunsaturated 2.5g	
MonounSaturated Fat 1g	
**Cholesterol** 0mg	**0**%
**Sodium** 180mg	**8**%
**Potassium** 110mg	**3**%
**Total Carbohydrate** 19g	**6**%
Dietary Fiber 3g	**12**%
Sugars 0g	
**Protein** 3g	
Vitamin A 0% • Vitamin C 0%	
Calcium 0% • Iron 4%	
Phosphorous 10%	

INGREDIENTS: WHOLE WHEAT, SOYBEAN AND/OR PALM OIL, SALT. **CONTAINS: WHEAT.**

Serving per container lists how many portions per container.

% Daily value is based on a 2,000 calorie daily diet. This number helps you know if a food is high or low in a nutrient, even if you eat more than 2,000 calories.

Potassium is not required to be listed by law. It is listed here, but it may not be listed even if the product contains potassium.

Ingredients are listed in order by weight, with the item of the most weight listed first. If potassium chloride is in the ingredient list, it has a high potassium content.

In general, % of potassium means:

- Low = Under 100 mg or less than 3%
- Medium = 101–200 mg or 3–6%
- High = 201–300 mg or 6–9%
- Very High = Over 300 mg or over 9%

Use healthy tips

to shop, plan and prepare meals with less potassium

At home:

- Don't drink or use liquid from canned fruits, vegetables, or cooked meat.
- Eat a variety of foods, but in moderation.
- Serving size is very important. Almost all foods have some potassium. A large amount of a low-potassium food can turn into a high-potassium food.

At restaurants

- Choose restaurants best suited to your diet or where food is made to order. Call ahead and explain that you are following a special diet. Ask about the menu and how their food is prepared. Many restaurants have websites with menus posted.
- To have more choices when eating out, avoid higher-potassium fruits and vegetables during the day beforehand.
- Choose starches and vegetables that are lower in potassium (such as rice, noodles and green beans).
- Avoid french fries and other fried potatoes.
- For desserts:
  - Choose desserts with simple preparations to avoid "hidden" phosphorus and potassium.
  - Avoid desserts with chocolate, cream cheese, ice cream or nuts – they are high in potassium and phosphorus.

Ethnic foods

For Chinese food:

- Choose lower-potassium vegetables such as snow peas, string beans, water chestnuts, bean sprouts and bok choy.

For Mexican food:

- Be cautious – Mexican food may be high in potassium.
- Beware of salsa. Salsa verde is a green sauce but is made of green tomatoes. Best choices: salsas made of chili peppers without tomatoes added.
- Avoid beans and rice. Both are high in potassium.
- Avoid guacamole. It is made from avocados, which are high in potassium.

For Italian food:

- Avoid tomato sauces. Tomatoes are high in potassium, but pasta is served with many sauces that are not tomato-based. If you must select a dish with tomato sauce, ask that it be served on the side.

For Soul food:

- Be cautious – it is often high in potassium.
- Avoid black-eyed peas, dried beans, cooked greens, spinach, yams, and sweet potato pie. All are high in potassium.
- Best choices? Fried chicken (no skin), corn, string beans or okra, wilted lettuce, corn bread or dinner rolls.

© 2009 National Kidney Foundation, Inc. All rights reserved. 02-10-0410_JAJ

If you need to limit

potassium

Potassium is a mineral that helps keep your heart and muscles working properly.

Why limit potassium?

Having too much potassium in your blood can cause:

- heart attack
- muscle weakness
- irregular heartbeat

How much is okay to eat?

Your doctor recommends eating less than

________ mg per day.

PARTNERS IN EDUCATION

National Kidney Foundation®

LitholinkCKD
Chronic Kidney Disease Management Program™

What are high-potassium foods?

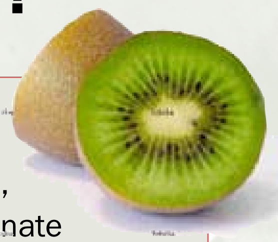

Fruits

- Bananas, melons, oranges, nectarines, kiwi, mango, papaya, prunes, pomegranate
- Dates, dried fruits, dried figs

Vegetables

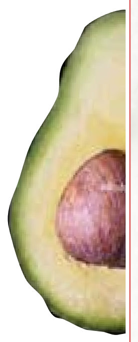

- Avocados, broccoli, brussels sprouts, sweet potatoes, parsnips, pumpkin, vegetable juices, white potatoes, winter squash
- Tomato and tomato-based products
- Deep-colored and leafy green vegetables (such as spinach or swiss chard)
- Dried beans and peas, black beans, refried beans, baked beans, lentils, legumes

Other

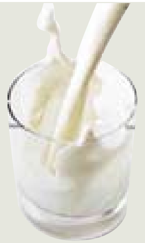

- Milk, yogurt
- Nuts and seeds
- Bran and bran products
- Chocolate, granola, molasses, peanut butter
- Salt substitutes

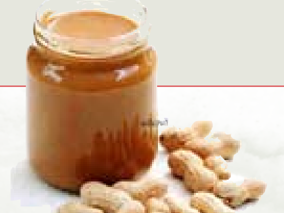

What are lower-potassium foods?

Fruits

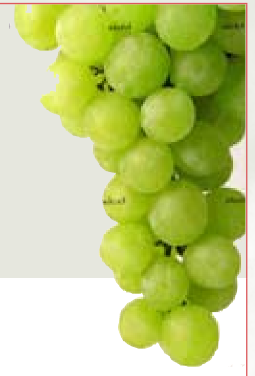

- Apple, blueberries, cranberries, grapes, grapefruit, peaches, pears, pineapple, raspberries

Vegetables

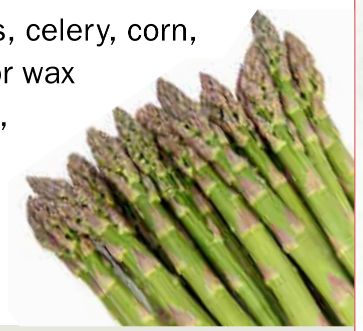

- Asparagus, cabbage, carrots, celery, corn, cucumber, eggplant, green or wax beans, green peas or beans, lettuce (iceberg), onions, radishes, turnips, water chestnuts

Other

- Rice, noodles, pasta, bread and bread products (not whole grains)
- Angel cake, yellow cake, pies without chocolate or high-potassium fruit, cookies without nuts or chocolate

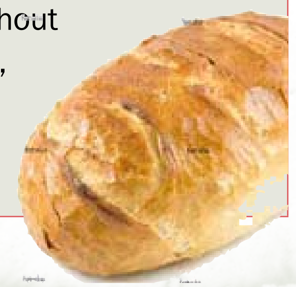

What is a common serving size?

FOOD GROUPS	SERVING SIZES
**Meat, fish or poultry**	
Meats, poultry, fish	1 oz cooked
**Dairy**	
Milk or milk substitute	4 oz or 1/2 cup
Egg	1 egg<br>or 1/4 cup egg substitute
Cheese	1 oz
**Grains**	
Cooked pasta, rice	1/3 cup
Cereal, cooked	1/2 cup
Cereal, ready to eat	1 cup
Bread	1 slice
Hamburger bun	1/2 bun
**Vegetables**	
Cooked	1/2 cup
Raw	1 medium or 1 cup cut-up
Juices	4 oz or 1/2 cup

Dietitians who specialize in kidney disease can tell you how many servings from each food group you are allowed at each meal. Depending on your diet prescription and preferences, you may be allowed to eat more than one.

FOOD GROUPS	SERVING SIZES
**Fruits**	
Fresh	1 small or 1/2 large
Canned or frozen fruit	1/2 cup
Juices	4 oz or 1/2 cup
Berries	1 cup
Grapes, cherries	12
Dried fruit	1/4 cup
**Fats and oils**	
Oils, margarine	1 teaspoon
Mayonnaise	1 tablespoon
Salad dressing	2 tablespoons
**Sweets**	
Cookie	1 cookie
Ice cream, sorbet, gelatin	1/2 cup
Cake, 2 x 2 inches	1 piece
Fruit pie	1/6 of 8-inch pie
Sugar, jelly, jam	1 tablespoon
**Nuts, seeds, and legumes**	
Nuts	1/4 cup or 1 oz
Seeds	2 tablespoon
Legumes, dry beans, peas, cooked	1/2 cup
Peanut butter	2 tablespoon
